# Supplementary figures and images for: Effect of SGLT-2 inhibitors on body composition in patients with type 2 diabetes mellitus: A meta-analysis of randomized controlled trials
Source: PLoS One. 2022 Dec 30;17(12):e0279889. doi: 10.1371/journal.pone.0279889 (PMC9803203; doi:10.1371/journal.pone.0279889)

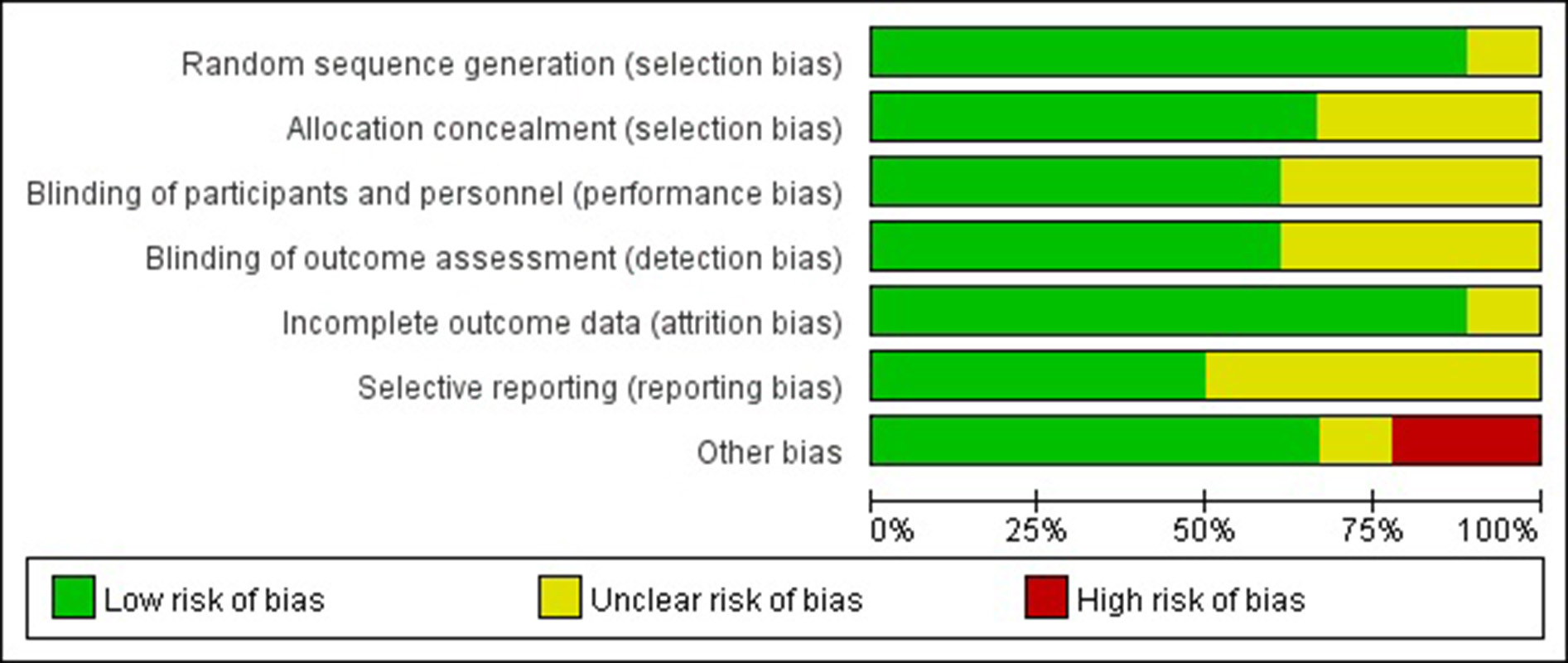

Supplement: S1 Fig — (TIF) [file pone.0279889.s001.tif]

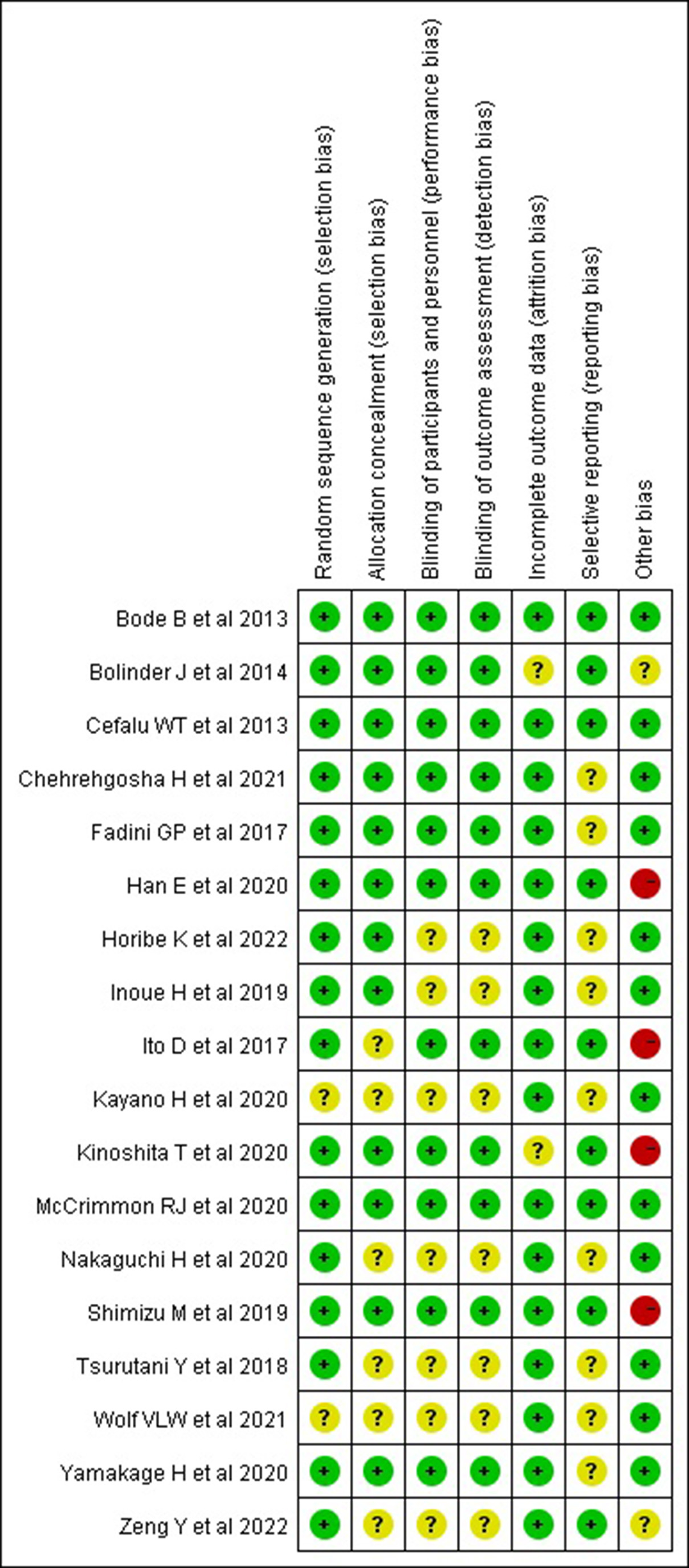

Supplement: S2 Fig — (TIF) [file pone.0279889.s002.tif]

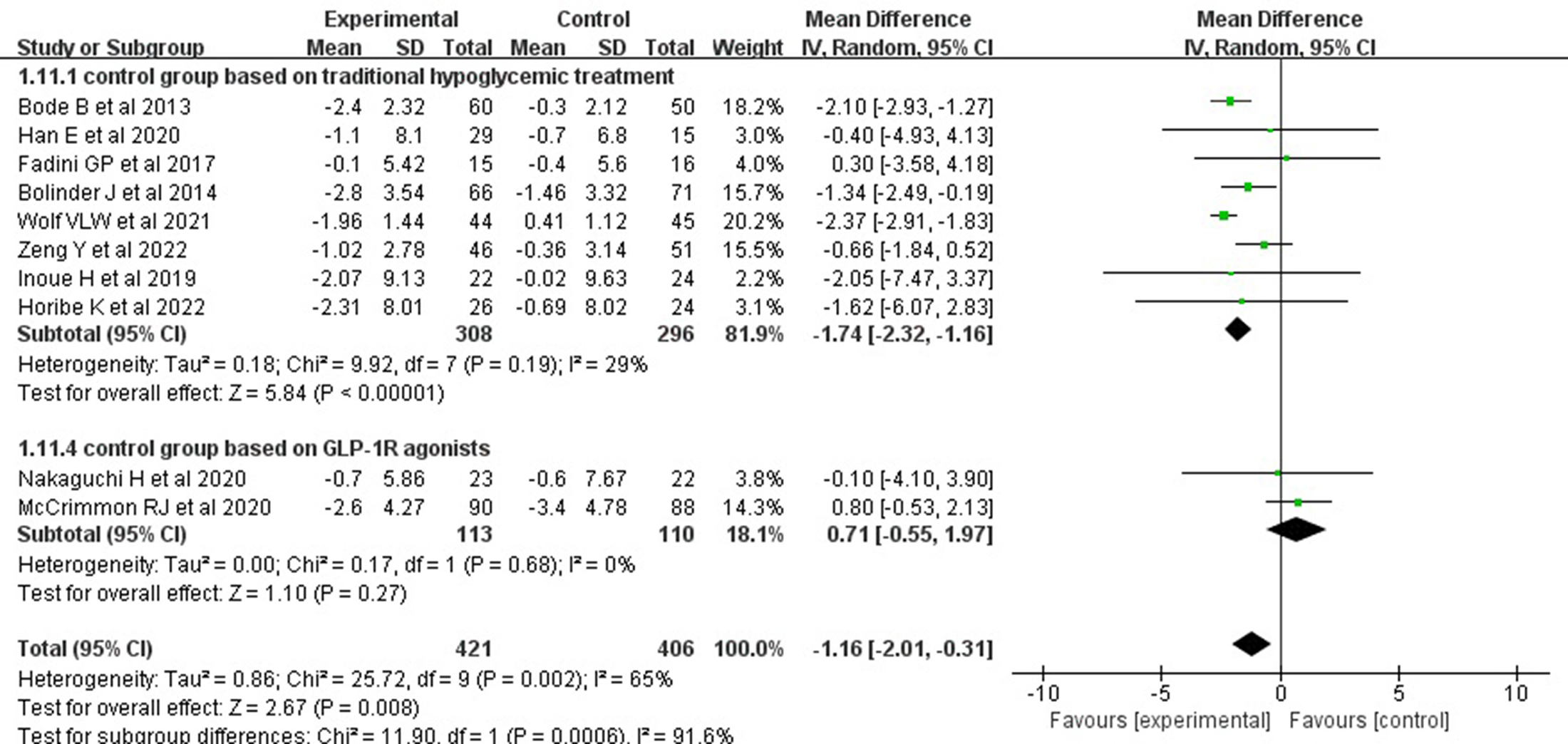

Supplement: S3 Fig — (TIF) [file pone.0279889.s003.tif]

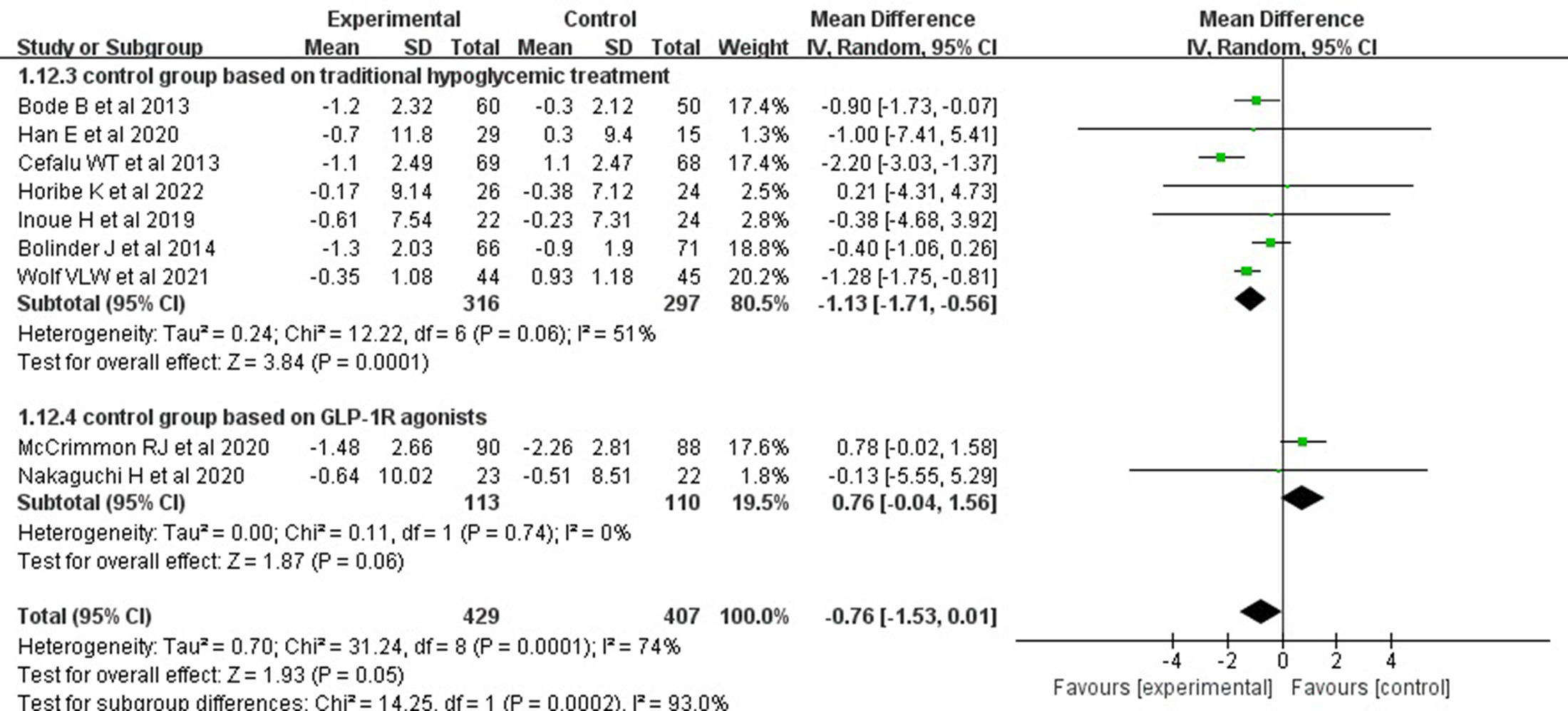

Supplement: S4 Fig — (TIF) [file pone.0279889.s004.tif]
