# Supplementary material for: Effect of SGLT-2 inhibitors on body composition in patients with type 2 diabetes mellitus: A meta-analysis of randomized controlled trials
Source: PLoS One. 2022 Dec 30;17(12):e0279889. doi: 10.1371/journal.pone.0279889 (PMC9803203; doi:10.1371/journal.pone.0279889)
Supplement: Attachment — Submitted filename: Response to Reviewers.docx [file pone.0279889.s006.docx]

Gulali Aktas

Editor-in-Chief

PLOS ONE

2022-12-03

Dear Dr Aktas

Please find attached a revised version of our manuscript“Effect of SGLT-2 inhibitors on body composition in patients with type 2 diabetes mellitus:A meta-analysis of randomized controlled trials”(No:PONE-D-22-30100) which we would like to resubmit for publication as a research article in PLOS ONE.

Your comments and those of the reviewers were highly insightful and enabled us to greatly improve the quality of our manuscript. In the following pages are our point-by-point responses to each of the comments of the reviewers as well as your own comments.

Revisions in the manuscript are shown using “Track Changes” option in the"Revised Article with Changes Highlighted” file.In accordance with Journal requirements,style modifications were made to ensure that the manuscript meets PLOS ONE's style requirements(e.g.We updated and replaced Fig 1).We have checked the reference list and made sure it is complete and correct.We made changes in accordance with two reviewers' suggestion,shown using “Track Changes” option in the manuscript.

We hope that the revisions in the manuscript and our accompanying responses will be sufficient to make our manuscript suitable for publication in Plos One.

We shall look forward to hearing from you at your earliest convenience.

Yours sincerely,

Yongcai Zhao Dr.

Address: Department of Endocrinology,Cangzhou Central Hospital,Cangzhou,Hebei Province,China

Email:yongcaizhao2022@163.com

****Responses to the comments of Reviewer #1****

Abstract is enough, The introduction is clear and explanatory. material method, statistics part is well explained and designed. The findings are beautifully and clearly written, both in writing and figures. In the literature, studies related to sglt2 have been scanned and discussed in a nice and detailed way. The discussion section could be expanded a bit though. You can benefit from the article mentioned in the journal below. I would also expect an important side effect such as euglycemic diabetic ketoacidosis to be mentioned in the discussion. In this respect, the article in the journal number 2 can be used.
1.Irish Journal of Medical Science (1971-), 191(4)
2.Journal of the College of Physicians and Surgeons--Pakistan: JCPSP, 32(7)

**Response:**

*I have accepted all your suggestions and add the following in the discussion section:In addition,the weight loss caused by SGLT-2 inhibitors may be due in part to body water loss**,based on a unique glucose-lowering mechanism^[38]^.However,it should be noted that studies of ketosis and euglycemic ketoacidosis based on dehydration and insulinopenia during SGLT-2 inhibitors use have been reported^[34,39,40]^.Due to limited data,we did not conduct further analysis of water loss and risk of* *ketosis caused by SGLT-2 inhibitors.However,dehydration and ketosisis are also potential adverse effects that needs to be considered during SGLT-2 inhibitors use.*

*References*

*[38] Post A, Groothof D, Eisenga MF, Bakker SJL. Sodium-Glucose Cotransporter 2 Inhibitors and Kidney Outcomes: True Renoprotection, Loss of Muscle Mass or Both? J Clin Med.2020. 9(5): 1603. DOI: 10.3390/jcm9051603. PMID: 32466262.*

*[39] Bilgin S, Duman TT, Kurtkulagi O, Yilmaz F, Aktas G. A Case of Euglycemic Diabetic Ketoacidosis due to Empagliflozin Use in a Patient with Type 1 Diabetes Mellitus. J Coll Physicians Surg Pak. 2022. 32(7): 928-930. DOI: 10.29271/jcpsp.2022.07.928. PMID: 35795946.*

*[40] Bilgin S, Kurtkulagi O, Duman TT, Tel BMA, Kahveci G, Kiran M, et al. Sodium glucose co-transporter-2 inhibitor, Empagliflozin, is associated with significant reduction in weight, body mass index, fasting glucose, and A1c levels in Type 2 diabetic patients with established coronary heart disease: the SUPER GATE study. Ir J Med Sci. 2022. 191(4): 1647-1652. DOI: 10.1007/s11845-021-02761-6. PMID: 34476725.*

****Responses to the comments of Reviewer #2****

Dear author, the meta-analysis 'Effect of SGLT-2 inhibitors on body composition in patients with type 2 diabetes mellitus: A meta-analysis of randomized controlled trials' ;was evaluated. In this meta-analysis, the contribution of a group of drugs that are currently at the top of the preventive and therapeutic guidelines of all cardiovascular, diabetes and kidney diseases to muscle loss is evaluated.I believe that it will contribute to the literature because it is a remarkable point.
Limitation: The fact that the studies included in the meta-analysis are from different ethnic groups, age ranges, genders and patient groups with comorbidities, and that there are many parameters that may affect muscle loss suggest that it is not appropriate to evaluate the results only for SGLT-2 treatment. Pre-planned prospective control and large-scale studies will be more instructive in this regard.

**Response:**

*I have accepted all your suggestions and add the following in the discussion section:Fourth,the studies included in the meta-analysis are from different ethnic groups, age ranges, genders and patient groups with comorbidities, and that there are many parameters that may affect muscle loss,suggesting that pre-planned prospective control and large-scale studies will be more instructive in this regard.*
